# Effects of Cadmium Exposure on Growth and Metabolic Profile of Bermudagrass [*Cynodon dactylon* (L.) Pers.]

**DOI:** 10.1371/journal.pone.0115279

**Published:** 2014-12-29

**Authors:** Yan Xie, Longxing Hu, Zhimin Du, Xiaoyan Sun, Erick Amombo, Jibiao Fan, Jinmin Fu

**Affiliations:** 1 Key Laboratory of Plant Germplasm Enhancement and Specialty Agriculture, Wuhan Botanical Garden, Chinese Academy of Sciences, Wuhan City, Hubei, People's Republic of China; 2 Graduate University of Chinese Academy of Sciences, Beijing, People's Republic of China; Korea University, Republic of Korea

## Abstract

Metabolic responses to cadmium (Cd) may be associated with variations in Cd tolerance in plants. The objectives of this study were to examine changes in metabolic profiles in bermudagrass in response to Cd stress and to identify predominant metabolites associated with differential Cd tolerance using gas chromatography-mass spectrometry. Two genotypes of bermudagrass with contrasting Cd tolerance were exposed to 0 and 1.5 mM CdSO_4_ for 14 days in hydroponics. Physiological responses to Cd were evaluated by determining turf quality, growth rate, chlorophyll content and normalized relative transpiration. All these parameters exhibited higher tolerance in WB242 than in WB144. Cd treated WB144 transported more Cd to the shoot than in WB242. The metabolite analysis of leaf polar extracts revealed 39 Cd responsive metabolites in both genotypes, mainly consisting of amino acids, organic acids, sugars, fatty acids and others. A difference in the metabolic profiles was observed between the two bermudagrass genotypes exposed to Cd stress. Seven amino acids (norvaline, glycine, proline, serine, threonine, glutamic acid and gulonic acid), four organic acids (glyceric acid, oxoglutaric acid, citric acid and malic acid,) and three sugars (xylulose, galactose and talose) accumulated more in WB242 than WB144. However, compared to the control, WB144 accumulated higher quantities of sugars than WB242 in the Cd regime. The differential accumulation of these metabolites could be associated with the differential Cd tolerance in bermudagrass.

## Introduction

Cadmium (Cd) is a vast environmental pollutant resulting primarily from anthropogenic activities including agriculture, mining, metallurgy and manufacturing [1], [2]. It is highly mobile, bio-accumulated in lower organisms and transferred to higher trophic levels in the food chain. Therefore, Cd is considered by the US Environmental Protection Agency (EPA) as one of the three major contaminants of greatest threat to the environment (along with mercury and lead) [3]. Guangzhou Food and Drug Administration in China reported on May 16, 2013 that 44.4% sampled rice contained more Cd than national standards, Cd contaminated soils has therefore caused widespread public concerns and anxiety [4].

It has been postulated that higher plants are more sensitive to Cd stress. Although Cd is non-essential for plant growth, it is readily taken up by roots and translocated to the shoots. Therefore its presence in the environment, even at minimum quantities poses a severe threat to plants [5]. Previous studies have shown that Cd could decrease carbon assimilation, generate oxidative stress, inhibit chlorophyll synthesis, reduce nutrient uptake, impair photosynthesis and inhibit plant growth [6]–[10]. Larsson et al. (1998) and Baryla et al. (2001) reported that low Cd concentrations in *Brassica napus* reduces its growth rate, chlorophyll content and leads to stomatal closure which eventually results in the transpiration rate decline [10], [11]. It has been suggested that apart from plant growth retardation, Cd could have a direct effect on enzymes of the chlorophyll biosynthesis pathway [12]. Therefore, Cd uptake and accumulation in plants initiates a series of morphological, physiological and biochemical changes.

Cd toxicity also affects numerous metabolic processes in plants. Recently, metabolic profiling has been considered a powerful tool for the discovery of metabolites and metabolic pathways in the field of plant response to stress [13]–[15]. A series of metabolites, such as organic acids, amino acids, sugars and sugar alcohols may function as antioxidants, osmo protectants, by-products of stress as signal transduction molecules during environmental stress [16]–[18]. Amino acids are the constituents and are vital in Cd tolerance metabolism in plants. In response to toxic metals, plants accumulate specific amino acids, which may be beneficial to Cd tolerance of plant [19]–[22]. Previous studies confirmed that proline may accumulate under toxic metals stress and function as a protein-compatible hydrotrope [23]. Salt et al. (1999) reported that histidine play a major role in Zn homeostasis in the root and that organic acids are involved in Zn storage and xylem transport in the shoots of *Thlaspi caerulescens* [24]. In *Alyssum*, histidine accumulation is responsible for nickel hyperaccumulation [25]. Smirnoff and Stewart (1987) reported that asparagine could alleviate Zn toxicity in *Deschampsia cespitosa* by forming an intracellular complex with Zn [26]. Xu et al. (2012) found that Cd also markedly increased the production of several organic and amino acids in *S. nigrum* [22]. Exogenous citric acid and amino acid can improve Cd tolerance of Cd-treated *S. nigrum*. These findings suggested that the compaction of heavy metals by metabolites, such as organic acids and amino acids, is crucial in metal detoxification, transport and accumulation [22], [27]–[29]. However, how full metabolic profile respond to Cd stress has not been comprehended.

Bermudagrass [*Cynodon dactylon* (L.) Pers.] is one of the most globally used warm-season turfgrass species. Previous studies along with the authors' recent investigation (unpublished data) demonstrated that bermudagrass was the most dominant species in Cd contaminated soil and might have evolved heavy metal tolerance. Therefore, bermudagrass has the potential to be applied in revegetation of contaminated soil [30], [31]. Fully-developed rhizome of bermudagrass can form compact ground cover that effectively prevents Cd loss and spread caused by rainfall. Previous studies indicated that different types of metabolites may accumulate in response to abiotic stress in different plant species, depending on the species and the severity of stress in different studies [32]. Cd-tolerant plants exhibit a number of biochemical changes leading to Cd-induced production of organic acids, such as acetic acid, malic acid, citric acids and L-tartaric acid which could desorb heavy metals from the soil matrix into the soil solution and facilitate metal transport into the xylem. This process may promote the translocation of heavy metals from the roots to the shoots [27], [33]–[35]. However, the mechanism of tolerance to Cd induced metabolic profile in bermudagrass which is a potential selection criterion for improving plant Cd tolerance has not yet been thoroughly investigated in bermudagrass. The objectives of this study were to characterize the impact of Cd on Cd-tolerance and sensitive bermudagrass growth parameters and plant fitness, to examine metabolites in response to Cd stress using gas chromatography–mass spectrometry (GC-MS) analysis, to identify predominant metabolites responsive to Cd stress in bermudagrass, and to compare Cd tolerance and accumulation between tolerant and sensitive bermudagrass genotypes.

## Materials and Methods

### Plant materials and growth conditions

Single stem section of 2 bermudagrass genotypes with contrasting Cd tolerance, WB242 (tolerant) and WB144 (sensitive) [36], was allowed to propagate vegetatively in plastic pots (15 cm in diameter and 20 cm tall) filled with solid growth substances (3 sand: 1 peat soil, v/v). The pots were placed in a greenhouse for 3 months to allow the establishment of roots and shoots. During growth period, plants were irrigated 3 times per week and fertilized weekly with 50 mL of half strength Hoagland's solution [37]. The grasses were hand clipped to an 8 cm height every week. After 3 month establishment, the plants were rinsed thoroughly using distill water and transplanted into Erlenmeyer flasks (300 mL in volume) filled with 280 mL of half-strength Hoagland's solution with MgO_2_, which provided oxygen, and refilled with fresh nutrient solution every 2 days. The flasks were wrapped with aluminum foil to prevent potential growth of algae, the bottlenecks were sealed using an appropriate size of absorbent paper covered by preservative film to prevent moisture loss. All flasks were placed in the growth chamber with standard conditions (30/25°C for day/night, 14 h light/10 h dark, 400 umol photons m^−2^ s^−1^ of light intensity, 70±10% relative humidity) for adaptation for 2 weeks. And the grasses were hand clipped to an 8 cm height every week during adaption period.

### Treatments and experimental design

After 2 weeks adaptation, transpiration rate (TR) was measured by the difference in the plant-flask system weight on a 24 h interval. Plant-flask system with similar TR were grouped the same replication in order to reduce the initial differences. Bermudagrass was exposed to 0 (*i.e.* control, half-strength Hoagland solution) and 1.5 mM Cd (CdSO_4_.8/3H_2_O dissolved in half-strength Hoagland solution). The containers with plants were kept in the above-mentioned conditions for 14 d. Genotypes and treatments were arranged in a completely randomized design with four replicates.

### Measurements

Turf quality (TQ) was assessed visually on a weekly basis using a scale of 0 to 9, in which 0 is withered, yellow and dead grass, 9 indicates green, dense and uniform grass and 6 is the minimum acceptable level according to density, uniformity, and color [38].

To determine growth rate (GR), all plants were trimmed to 8 cm canopy height before treatment. A sheet of filter paper was twisted to a tube which can rested on the Erlenmeyer flask bottle ensure the plants were cut to equal height each week. Fresh weight of clippings was recorded. GR was estimated by measuring the difference in fresh weight of leaves collected between two cuttings in 24 h [39].

The leaf chlorophyll (Chl) content was measured using the method described by Hiscox and Israelstam (1979) [40]. Briefly, fresh leaves (0.1 g) were sheared into small pieces and soaked in 15 mL-tubes within 10 mL dimethylsulfoxide to extract Chl under dark conditions for 72 h. The absorption of samples at 645 and 663 nm was measured on a spectrophotometer (UV-2600, UNICO, Shanghai). The content of Chl a, Chl b and Chl a+b were calculated by the equations described by Hiscox and Israelstam (1979).

To access normalized relative transpiration, the plant-flask containers were weighted every 48 h to determine transpiration rate by recording absolute transpiration. Absolute transpiration was normalized to initial and non-contaminated transpiration which made data comparable across all sample. The mean normalized relative transpiration (NRT) was formulated as Eq. (1) 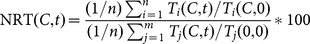



In Eq. (1), T indicates absolute transpiration (g d^−1^), t is recording interval (0–48, 48–96 h, etc.), C represents Cd concentration (mg L^−1^), *i* and *j* represents replicates of Cd stressed category and control category respectively [41]. NRT value less than 100% indicates inhibition effect on transpiration while above 100% means stimulation.

### Inductively coupled plasma mass spectroscopy (ICP-MS) Analysis

Cd accumulation was accessed using inductively coupled plasma mass spectroscopy (ICP-MS). At the end of the experiment, the roots and shoots were harvested separately and immersed in 10 mM Na_2_EDTA for 1 h and then rinsed thoroughly with distilled water 3 times to remove any nonspecifically bound Cd. The samples were oven-dried at 75°C for 48 h. The oven-dried plant tissues were ground into fine powder and then subsamples of ground shoot samples (100 mg) and root samples (30 mg) were digested in a mixture of concentrated HNO_3_ and HClO_4_ (4∶1, v/v). The volume of each sample was adjusted to 50 mL using double deionized water. The concentration of Cd was determined using ICP-MS. Each experiment was repeated at least 3 times. The translocation factor (TF) of Cd from root to shoot was calculated as follows [42]: 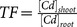 Eq. (2).

### Metabolite extraction and derivation

Plant samples for metabolite assay were harvested from bermudagrass leaves after treatments. Fully expanded leaves (0.1 g) were collected and immediately frozen in liquid nitrogen and stored at −80°C until for analysis. The metabolite extraction and sample derivatization was modified from described previously [17], [43]. For each sample, frozen leaves were grounded to a fine powder with liquid nitrogen, powders were transferred into 2 mL microcentrifuge tubes and extracted in 1.4 mL of 80% (v/v) aqueous methanol for 2 h under 200 rpm at ambient temperature. Ten µL ribitol solution (2 mg mL^−1^ water) was added as an internal standard prior to incubation. The mixture was done in a water bath at 70°C for 15 min. The extraction solution was centrifuged for 10 min at 10,000 g, supernatant was decanted to new 10 mL tubes and 1.5 mL of water and 0.75 mL of chloroform were added. The mixture was vortexed thoroughly for 15 s and centrifuged for 10 min at 10,000 g. The 0.3 mL polar phase was decanted into 2 mL HPLC vials and dried in a Centrivap benchtop centrifugal concentrator overnight (Labogene, Denmark). The dried polar phase was methoximated with 80 µL of 20 mg mL^−1^ Methoxyamine hydrochloride at 30°C for 2 h followed by trimethylsilyl with 50 µL MSTFA for 2 h at 37°C. Standards and reagents were purchased from Sigma-Aldrich Co.Ltd. (Poole, UK).

### Gas chromatography mass spectrometry (GC-MS) analysis

The metabolites were determined using GC-MS (Agilent 7890A/5975C, Agilent Technologies, Palo Alto, CA, USA) by Qiu et al. (2007) protocol [44]. For GC-MS, 1 µL of derivatizated extract was injected into a DB-5MS capillary (30 m×0.25 mm×0.25 µm, Agilent J&W GC column, USA). The inlet temperature was set at 280°C. After a solvent delay for 5 min, initial GC oven temperature was set at 70°C; after injection for 1 min, the GC oven temperature was raised to 280°C with 5°C per min, and held at 300°C for 10 min. The injection temperature was set to 280°C and ion source temperature was matched. Helium was used as the carrier gas with a constant flow rate set at 1 mL min^−1^. The measurement was made with electron impact ionization (70 eV) in the full scan mode (m/z from 30 to 650).

### Metabolite data processing and analysis

The metabolites were identified based on retention time using the Agilent MSD Productivity Chemstation software coupled with commercially available compound libraries (NIST 11) (Gaithersburg, MD, USA). The relative quantification of metabolites was performed based on the internal standard ribitol. The hierarchical clustering analysis (HCA) and principal component analysis (PCA) were performed using the MetaboAnalyst webpage (http://www.metaboanalyst.ca/MetaboAnalyst/) [45]. Before statistical assessment, log-transformed response ratios were calculated for each identified metabolites.

### Statistical analysis

Significance of differences in metabolite ratios or other parameters when comparing different genotypes and treatment groups were assessed using one-way ANOVAs combined with Tukey tests at 0.05 level of probability (SAS Institute Inc., Cary, NC). Further metabolite analyses found that there was no significant interaction between Cd-tolerant and sensitive genotype.

## Results

### Differential physiological response of genotypes to Cd stress

**Figure 1:**
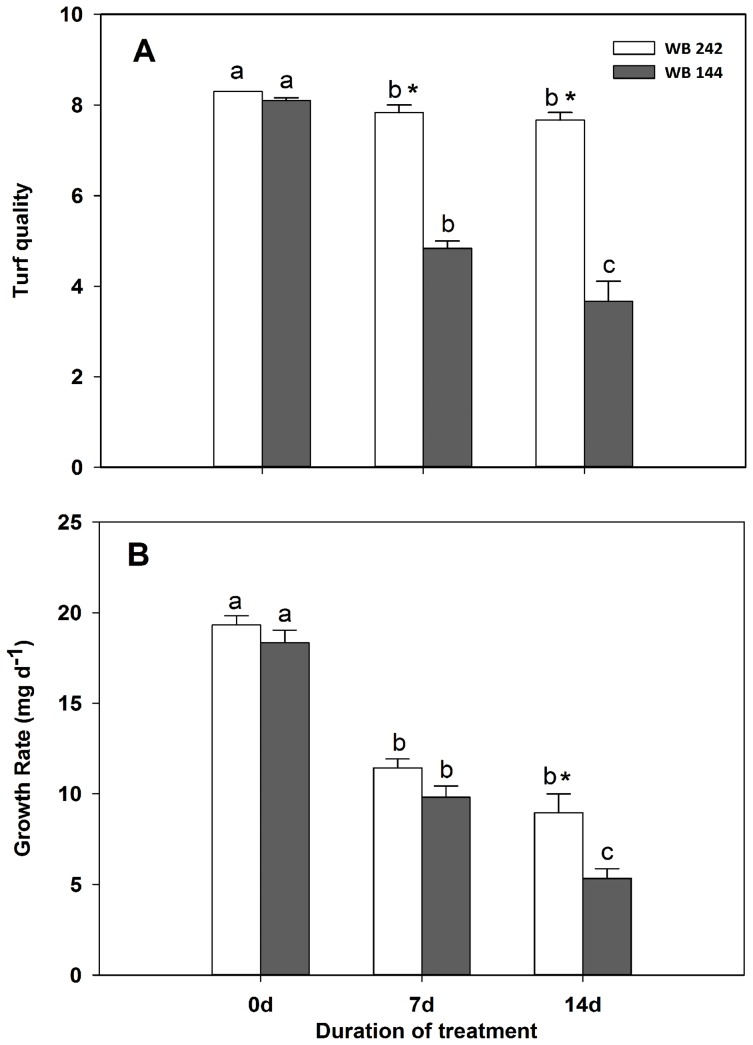
Effects of Cd stress on turf quality (A) and growth rate (B) for bermudagrass at 0, 7 and 14 d. Vertical bars indicate standard error of each mean. Columns marked with small letter indicate significant differences between d of treatment based on Tukey's test (*P*<0.05). Columns marked with star represent statistical significance for comparison between genotypes at a given day of treatment (*P*<0.05).

**Figure 2:**
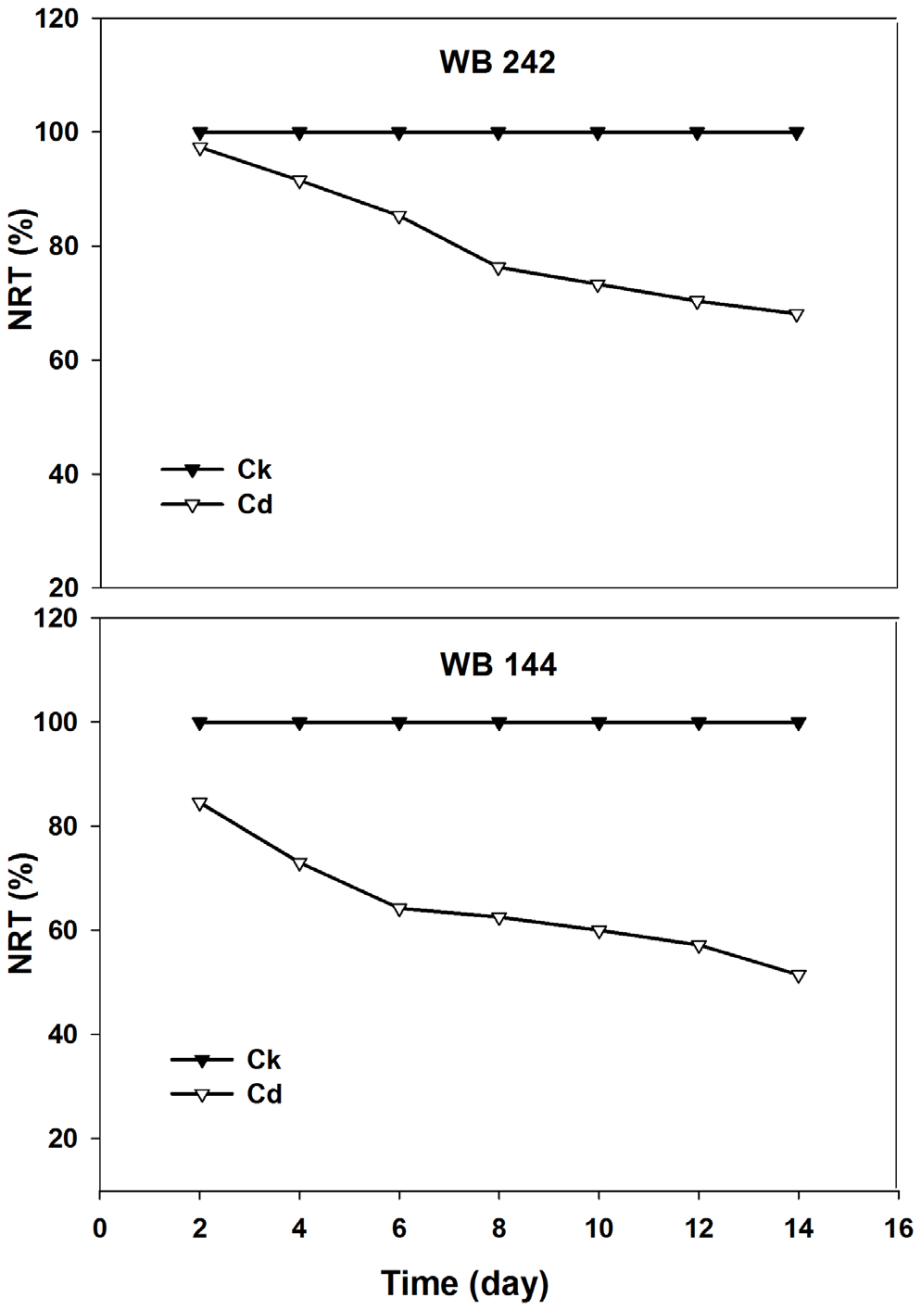
Normalized relative transpiration (NRT) of two genotypes in response to Cd stress.

As shown in Fig. 1, TQ and GR declined with Cd stress duration in the two bermudagrass genotypes, to a greater extent in Cd-sensitive WB144 than that of Cd-tolerant WB242. After samples were subjected to Cd stress for 7 or 14 d, turf quality was higher in WB242 than WB144 (Fig. 1A). Cd treatment inhibited the GR, and the inhibitory effect was more conspicuous in WB144. The GR decreased by 50.5% and 79.9% at 7 and 14 d of Cd stress for WB144, while decreased by 44.5% and 60.5% for WB242, respectively, when compared to the initial value (0 d stress) (Fig. 1B). Cd stress resulted in a lower level of NRT in the two bermudagrass genotypes during whole experimental period, to a higher reduction in WB144 than WB242 (Fig. 2).

After 7 and 14 d of Cd stress, Chl a, Chl b and Chl a+b content significantly decreased for WB144, when compared to the control (Table 1). Cd stress had no observable effects on Chl content of WB242. When subjected to Cd stress for 14 d, WB242 leaves contained more Chl a and Chl b than WB144. Total Chl content was higher for WB242 than WB144 at 14 d of treatment (Table 1).

**Table 1:** Chlorophyll content in bermudagrass exposed to Cd stress (0, 1.5 mM).

	Chl a (mg g-1 FW)		Chl b (mg g-1 FW)		Chl a+b (mg g-1 FW)	
	WB242	WB144	WB242	WB144	WB242	WB144
0 d	3.31 aA	3.35 aA	0.54 aA	0.53 aA	3.85 aA	3.88 aA
7 d	3.12 aA	2.09 bA	0.41 aA	0.35 bA	3.53 aA	2.44 bB
14 d	2.93 aA	1.33 bB	0.35 aA	0.18 cB	3.28 aA	1.51 bB

Means followed by the same lower-case letter in a column and followed by the same upper-case letter in a row for a given chlorophyll were not significantly different based on Tukey's test (*P*<0.05).

### The metabolic response to Cd stress

**Figure 3:**
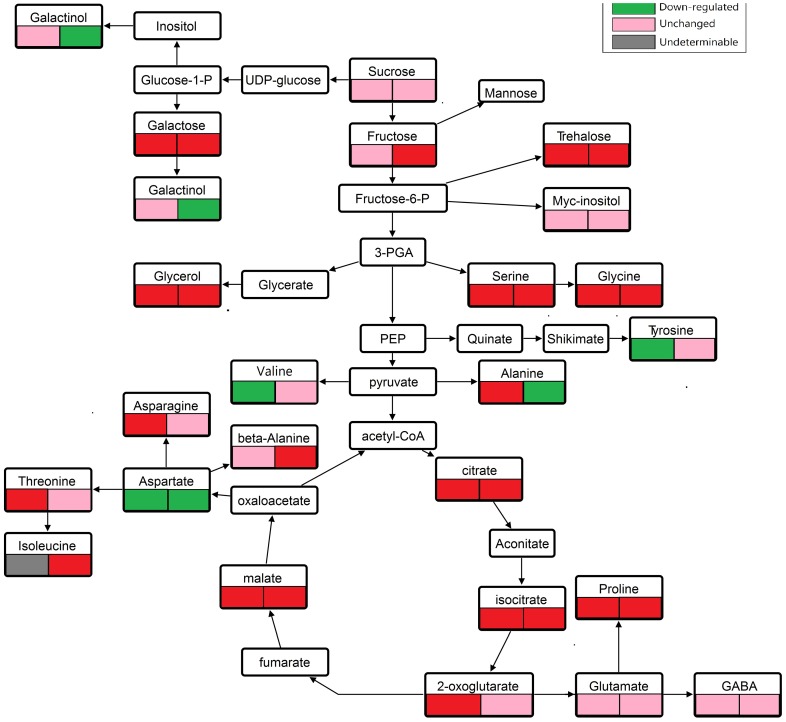
Metabolic pathways for metabolite shift in leaves under Cd stress in bermudagrass (corresponding to Table 2). Metabolites that have significantly higher or lower abundance in Cd-treatment leaves of WB242 (left) or WB144 (right) compared with their untreated control (*P*<0.05) are represented by red (higher) and green (lower) boxes. The pink boxes represent metabolites whose abundance is unchanged. The gray boxes represent metabolites concentrations that are undeterminable.

Totally, 39 metabolites were identified in the two bermudagrass genotypes (Table 2). The identified metabolites included amino acids (16), organic acids (7), sugars (8), fatty acids (3) and others (5) (including sugar alcohols, glycosides and amine). Following exposure to Cd conditions, WBD242 had a higher level of metabolites (Table 2, Fig. 3).

**Table 2:** Metabolite levels in leaves of bermudagrass exposed for 14 d to different concentrations of Cd (0, 1.5 mM).

Metabolites	WB 242		WB 144	
	Ck	Cd	Ck	Cd
**Amino acids**				
Alanine	0.074 c	0.106 b	0.360 a	0.168 b
Valine	0.066 a	0.024 c	0.034 bc	0.043 b
Serine	0.031 bc	0.088 a	0.017 c	0.039 b
Threonine	0.066 b	0.098 a	0.033 c	0.031 c
Isoleucine	UD	UD	0.010 b	0.024 a
Glycine	0.043 b	0.072 a	0.030 c	0.049 b
β-Alanine	0.006 b	0.006 b	0.024 b	0.113 a
GABA	0.042 b	0.068 a	0.035 b	0.025 c
Proline	0.027 c	0.174 a	0.024 c	0.062 b
Aspartic acid	0.251 a	0.190 b	0.263 a	0.113 b
Asparagine	0.026 b	0.077 a	0.084 a	0.070 a
Norvaline	0.145 ab	0.174 a	0.102 ab	0.086 b
Glutamic acid	0.595 a	0.494 a	0.394 b	0.419 b
Tyrosine	0.035 a	UD	0.008 b	0.007 b
Gulonic acid	0.027 b	0.074 a	0.007 c	0.038 b
Threonic acid	0.031 b	0.036 b	0.061 ab	0.079 a
**Sugars**				
Xylulose	0.307 a	0.210 a	0.032 b	0.027 b
Fructose	0.217 b	0.389 b	0.259 b	0.979 a
Galactose	0.202 b	0.514 a	0.094 c	0.322 b
Talose	0.328 c	1.368 a	0.164 c	0.845 b
Glucoheptose	0.053 b	0.264 a	0.055 b	0.229 a
Trehalose	0.027 c	0.139 b	0.132 b	0.217 a
Sucrose	12.934 a	13.178 a	21.602 a	23.130 a
Cellobiose	0.361 a	0.486 a	0.596 a	0.251 a
**Organic acids**				
Oxalic acid	0.421 b	0.710 a	0.404 b	0.548 a
Succinic acid	0.010 b	0.020 a	0.007 b	0.017 a
Glyceric acid	0.086 b	0.146 a	0.094 b	0.090 b
Oxoglutaric acid	1.764 b	2.127 a	0.008 c	0.022 c
Isocitric acid	1.661 b	1.593 c	3.130 a	2.283 b
Citric acid	2.526 b	4.685 a	1.356 c	2.004 b
Malic acid	1.837 c	3.948 a	1.616 c	2.064 b
**Fatty acids**				
Hexadecanoic acid	0.206 a	0.265 a	0.217 a	0.199 a
Octadecanoic acid	0.136 a	0.163 a	0.259 a	0.242 a
Glycerol	0.276 b	0.472 a	0.276 b	0.484 a
**Others**				
Myo-Inositol	0.270 a	0.195 a	0.205 a	0.229 a
Glyceryl-glycoside	0.556 b	0.570 b	1.059 a	0.035 c
Galactinol	0.820 a	0.627 a	0.693 a	0.341 b
Phosphate	0.052 a	0.057 a	0.136 a	0.054 a
Ethanolamine	0.094 a	0.053 a	0.090 a	0.075 a

Means followed by the different letter in a row for each metabolite was not significantly different based on Tukey's test (*P*<0.05). UD, undeterminable.

Seven amino acids were up regulated, 3 down regulated, and 6 unchanged in Cd stressed WB242 compared to the untreated WB242. The most prominent trend was revealed by the up-regulated metabolites in WB242 particularly serine-2.8-fold increase, proline-6.4-fold increase and asparagine-3.0-fold increase. Cd stressed WB144 exhibited 6 higher amino acids, 8 unchanged ones, and 2 lower ones compared to the untreated WB144 (Table 2, Fig. 3). Under Cd stress conditions, serine, threonine, glycine, proline, norvaline, glutamic acid and gulonic acid was 2.3, 3.2, 1.5, 2.8, 2.0, 1.2 and 1.9 fold higher in WB242 vs. WB144, respectively.

Seven organic acids were detected in the two bermudagrass genotypes (Table 2). WB242 and WB144 had a higher (5 to 7 out of 7) or unchanged (0 to 2 out of 7) level of organic acids in the Cd regime than the control. Under the Cd stress conditions, organic acids generally were higher in WB242 than WB144. Specifically, WB242 had a 96.7 fold oxoglutaric acid, 1.7-fold oxalic acid, 2.34 fold citric acid, and 1.43 fold malic acid compared to WB144 (Table 2, Fig. 3).

Generally, both bermudagrass genotypes accumulated more (4 to 5 out of 8) or unchanged (3 to 4 out of 8) sugar when subjected to Cd stress, more in WB144 than WB242. Five out of 8 sugars (fructose-3.8-fold increase, galactose-3.4-fold increase, talose-5.2-fold increase, glucoheptose-4.2-fold increase and trehalose-1.8-fold increase) were accumulated more in Cd stressed WB144 than the untreated WB144 (Table 2, Fig. 3).

Three fatty acids and 5 other metabolites were quantified in two bermudagrass genotypes. There was generally no observable difference in the content of these 8 metabolites between the control and the Cd regime for two genotypes (Table 2).

### Hierarchical cluster analysis and principle component analysis of steady-state metabolite concentrations

**Figure 4:**
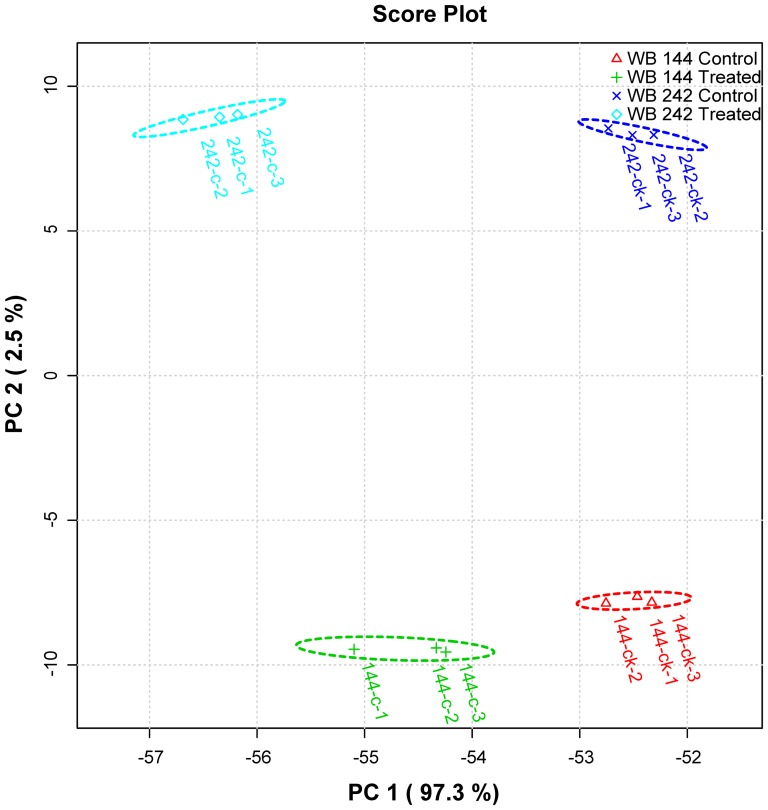
Principal Component analysis of the metabolites profiles in bermudagrass under Cd stress.

**Figure 5:**
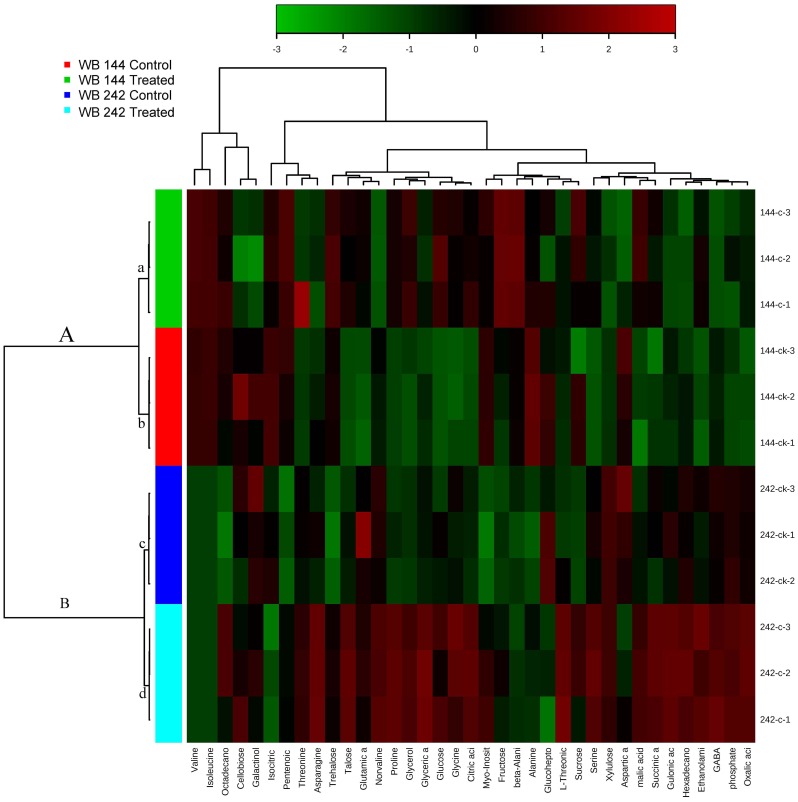
Hierarchical clustering analysis of metabolites affected by Cd treatment in bermudagrass.

Principal component analysis (PCA) analysis revealed a separation of the genotype subjected to Cd stress from control by the first dimension, which represented about 97.3% of the total variance (Fig. 4). The second principal component differentiated genotypes with about 2.5% of the variation. Simultaneously, the hierarchical cluster analysis (HCA) was applied, as shown in Fig. 5, it is convenient to find that all samples clustered into two major clusters corresponding to the two different genotypes (Cd-tolerant WB242 and Cd-sensitive WB144). Two subgroups corresponding to Cd stress and control could be distinguished with each other in the two major clusters.

### Cadmium accumulation and distribution

Bermudagrass shoots and roots had higher Cd concentrations at 14 d of Cd treatment regardless of genotypes compared to untreated plants (Table 3). There was no significant difference in Cd concentration between two untreated bermudagrass genotypes. Cd-tolerant genotype WB242 had a lower level of Cd concentration in shoot and higher Cd concentration in root than Cd-sensitively genotype WB144. Translocation factor of Cd-sensitive genotype WB144 was higher than Cd-tolerant genotype WB242 at the end of Cd treatment. However, under untreated conditions, a slightly higher translocation factor was observed in WB242 than WB144.

**Table 3:** Cd concentration (mg kg^−1^) in plant tissues and translocation factor (TF) in Cd-treated substrates for 14 d.

Treatment	Shoot		Root		TF	
	WB242	WB144	WB242	WB144	WB242	WB144
Ck	3.59 bA	3.61 bA	4.36 bA	5.19 bA	0.44 aA	0.37 aA
Cd	122.31 aB	154.19 aA	1082.37 aA	937.23 aB	0.11 bB	0.16 bA

Means followed by the same lower-case letter in a column and followed by the same upper-case letter in a row for a given tissue were not significantly different based on Tukey's test (*P*<0.05).

## Discussion

In this study, we investigated the effects of Cd stress on the plant growth and metabolic profiles in two genotypes of bermudagrass differing in Cd tolerance. Our results indicated that Cd exposure led to growth inhibition as indicated by the decrease in GR, TQ, NTR and Chl content. It has been reported that Cd stress disturbs physiological and metabolic processes, causes growth inhibition and a decrease in transpiration [46], [47]. However, Cd tolerant genotype WB242 exhibited a higher level of above parameters than Cd sensitive WB144. This finding was consistent with authors' previous study that WB242 was more tolerant to Cd toxicity than WB144 [36]. An important mechanism contributing to WB242 Cd tolerant might be its capacity to reduce the inhibition of photosynthesis and senescence. In plants, the most apparent symptom of Cd toxicity is chlorosis [7], [48]. Chloroplast structure can be damaged under Cd stress and the chlorophyll level decreased indirectly via metabolic perturbations and accelerated senescence [10], [49], [50]. The reduction in Chl content was detrimental to photosynthetic ability. Baryla et al. (2001) reported that the leaf chlorosis was attributable to a marked decrease in chloroplast density and suggested that Cd interfered with chloroplast replication [10]. The results of this study indicated that, Cd application resulted in a decrease in Chl content for both genotypes on a basis of fresh weight, the reduction in Chl content in Cd-sensitive WB144 was higher than in Cd tolerant WB242. Cd-tolerant WB242 contained higher concentrations of the Chl a, Chl b and total Chl under Cd stress compared to WB144, which suggested that WB242 has a high photosynthesis capacity under Cd stress conditions compared to WB144; this result may explain the higher TQ and NRT that were observed in WB242 in the presence of Cd. However, Cd stress had no observable effects on Chl content of WB242, but caused the decrease of growth rate of WB242. This result indicated that growth is highly sensitive to Cd stress, on the other hand, Cd specifically target the growth factors which are not related to photosynthesis for WB 242.

Cd accumulation differs remarkably within plant species and in the tissues [51]. Previous studies on Cd distribution showed that a big portion of the Cd taken up by the plant root was immobilized within the root tissues [52]. Sixty-five-90% of total Cd in the grasses was located in the roots [53], [54]. Thus the first line of defense against Cd toxicity, operating mainly at the root level, is the immobilization of Cd by means of the extracellular carbohydrates and cell wall [55], [56]. The authors' results indicated that Cd-tolerant genotype WB242 accumulated more Cd in the root, but less Cd in the shoot. Therefore, translocation factor of Cd-tolerant genotype WB242 was lower than WB144 after Cd treatment. It suggested that the Cd stress may be alleviated by sequestration and/or retention in the roots of Cd in WB242, which might be one of the reasons why WB242 was more Cd tolerant than WB144.

Amino acids are necessary for protein synthesis and serve as precursors for a large array of metabolites with multiple functions in response to various abiotic stresses. Cd stress also perturbed amino acid metabolism in plants [22]. The result of this study indicated that most amino acids were accumulated more in the leaves of WB242 than in the leaves of WB144. Under Cd stress conditions, WB242 leaves accumulated more proline than WB144 leaves. Proline has been well comprehended to be a stress marker [57], [58], an urgent osmoprotectant when plants are stressed to abiotic [22]. Xu et al. (2009, 2012) indicated that proline is vital in the alleviation of Cd toxicity by detoxifying ROS, thereby increasing the glutathione concentration and protecting antioxidative enzyme activities Cd accumulator and tolerant *Solanum nigrum* seedlings [22], [29]. Therefore, a higher accumulation of proline in WB242 supports the observed higher tolerance. The results of this study also exhibited that Cd stress reduced aspartic acid content, but did not affect glutamic acid content regardless of genotypes. This might be a consequence of buffering effects through modulations of threonine, asparagine, alanine, isoleucine and proline [22], [59]. These acids are direct or indirect products of aspartic acid and glutamic acid metabolism, respectively (Fig. 3). These results are consistent with previous studies in some species under Cd condition, such as Cd- accumulator *Solanum nigrum* [22] and tomato (*Lycopersicon esculentum*) [59]. The most remarkable changes observed are the increase of asparagine (3.0-fold) in WB242. Lea et al. (2007) has reported that asparagine was highly accumulated in toxic metal stressed plant [60]. Asparagine, an amide providing a high N:C ratio, could be used by the plant as a nitrogen storage compound to ensure future recovery [59]. Therefore, high asparagine levels could participate in the detoxification processes or by the biosynthetic way of chelating peptides [61]. Some secondary metabolites might be involved in stress defense, such as isoprenoid, phenylpropanoid, alkaloid or fatty acid/polypetide pathways [62]. Precursors of these compounds emanated from primary metabolism such as branched-chain amino acids (isoleucine and valine) serve as precursors for cyanogenic glycosides [63]. Xu et al. (2012) found accumulation of isoleucine and valine in *Solanum nigrum* (high Cd accumulation and tolerance) root under Cd stress [22]. However, our result showed that under Cd stress, isoleucine and valine contents increased for Cd-sensitive WB144 but unchanged for Cd-tolerant WB242, which suggests that two different stress defense pathway may exist in the two genotypes. Generally, metabolic analysis indicated the accumulation high content of amino acids in the leaf of WB242, which indicated high Cd tolerance. Therefore, a series of possible explanations exist for the beneficial effects of amino acid accumulation on Cd tolerance through participation in the detoxification processes by themselves or by the biosynthetic way of chelating peptide.

Organic acid compounds, such as isocitric acid, oxoglutaric acid, citric acid and malic acid which are crucial in the Krebs cycle, are often associated with heavy metal stresses and are considered to be a reliable indicators of Cd accumulation. The higher levels of Krebs cycle intermediates could be reflective of elevated mitochondrial activities which generate more reducing agents and ATP or for the provision of carbon skeletons for amino acid biosynthesis [64]. In this study, malic and citric acid concentrations of WB242 were higher than those in WB144, especially following Cd treatment. Several studies have indicated that organic acids may be vital in heavy metal tolerance [22], [65]. Sarret et al. (2002) found that *Arabidopsis halleri* contained high concentrations of malic acid, and Zn was predominantly compounded to malic acid in the shoots [66]. These observations suggest that protective mechanisms against Cd are activated rapidly to limit metal deleterious effects on leaves and the accumulation of organic acids can participate in the detoxification processes by chelating metal ions.

Cd toxicity also perturbed sugars metabolism in plants. Previous research has reported that sugar is necessary in plant growth and stress adaptation and is directly involved in the synthesis of other compounds, energy production, membrane stabilization, acting as regulators of gene expression and sugar-sensing signal system [67]–[70]. A recent study on the shoots of rice submitted to Cd shows that the accumulation of sugars was coupled with a decline of net photosynthetic rates [71]. Kieffer et al. (2008) reported that, the accumulation of various sugars (fructose, galactose, sucrose and glucose) could be one of the Cd effects leading to growth inhibition [72]. Our results indicated that, sugars, especially fructose, galactose, talose and glucoheptose increased in both genotypes, to a larger extent in Cd-sensitive genotype WB144 than Cd-tolerant genotype WB242. These results are consistent with the observation that WB242 grew better than WB144 under Cd stress. This suggested that, sugar utilization is reduced in the presence of Cd. Therefore photoassimilates are less needed for developmental processes and partially stored under some sugars [72], [73]. The accumulation of trehalose has been correlated with higher tolerance to a variety of abiotic stresses [74]. Garg et al. (2002) demonstrated that regulated overexpression of trehalose biosynthetic genes in rice has considerable potential for improving abiotic stress tolerance [75]. In this study, we observed a significant increase in the levels of trehalose in Cd-tolerant genotype WB242 (5.1-fold increase) while WB144 did not experience similar changes (1.8-fold increase), supporting a beneficial role for trehalose in bermudagrass during Cd stress.

In summary, physiological responses to Cd stress confirmed that WB242 exhibited better Cd tolerance than WB144. The differential Cd tolerance in these two genotypes could be attributed by differential adaptive response. The first line of defense against Cd toxicity for WB242 may be the immobilization of Cd by root. The metabolic profiling demonstrated that the differential Cd tolerance could be attributed by differential accumulation of amino acids (proline, aspartate, alanine, glycine, asparagine and serine), organic acids (citric acid, malic acid and oxalic acid) and sugars (trehalose, fructose, galactose and talose). These metabolites that mainly serve as intermediates in osmolytes, antioxidants and involved in other stress defense pathway may be play important roles in bermudagrass adaptation to Cd stress. However, how the Cd-responsive metabolites are directly correlated to Cd tolerance in bermudagrass requires further investigation through cellular and molecular level for better understanding of mechanisms of plants adaptation to Cd stress.

## Supporting Information

S1 Supporting Information **Overview of the metabolite reporting list.**
(XLSX)
